# Genome-wide association study identifies tumor anatomical site-specific risk variants for colorectal cancer survival

**DOI:** 10.1038/s41598-021-03945-x

**Published:** 2022-01-07

**Authors:** Julia D. Labadie, Sevtap Savas, Tabitha A. Harrison, Barb Banbury, Yuhan Huang, Daniel D. Buchanan, Peter T. Campbell, Steven J. Gallinger, Graham G. Giles, Marc J. Gunter, Michael Hoffmeister, Li Hsu, Mark A. Jenkins, Yi Lin, Shuji Ogino, Amanda I. Phipps, Martha L. Slattery, Robert S. Steinfelder, Wei Sun, Bethany Van Guelpen, Xinwei Hua, Jane C. Figuieredo, Rish K. Pai, Rami Nassir, Lihong Qi, Andrew T. Chan, Ulrike Peters, Polly A. Newcomb

**Affiliations:** 1 Public Health Sciences Division, Fred Hutchinson Cancer Research Center, Seattle, WA USA; 2 Department of Epidemiology, University of Washington, Seattle, WA USA; 3 Discipline of Genetics, Faculty of Medicine, Memorial University, St. John’s, NL Canada; 4 Discipline of Oncology, Faculty of Medicine, Memorial University, St. John’s, NL Canada; 5 Centre for Epidemiology and Biostatistics, Melbourne School of Population and Global Health, The University of Melbourne, Melbourne, VIC Australia; 6 Colorectal Oncogenomics Group, Genetic Epidemiology Laboratory, Department of Pathology, The University of Melbourne, Parkville, VIC Australia; 7 Genetic Medicine and Family Cancer Clinic, The Royal Melbourne Hospital, Parkville, VIC Australia; 8 Department of Population Science, American Cancer Society, Atlanta, GA USA; 9 Lunenfeld Tanenbaum Research Institute, Mount Sinai Hospital, University of Toronto, Toronto, ON Canada; 10 Cancer Epidemiology Division, Cancer Council Victoria, Melbourne, VIC Australia; 11 Medicine, School of Clinical Sciences at Monash Health, Monash University, VIC, Australia; 12 Nutrition and Metabolism Section, International Agency for Research On Cancer, World Health Organization, Lyon, France; 13 Division of Clinical Epidemiology and Aging Research, German Cancer Research Center (DKFZ), Heidelberg, Germany; 14 Program in Molecular Pathological Epidemiology, Department of Pathology, Brigham and Women’s Hospital, Harvard Medical School, Boston, MA USA; 15 Cancer Immunology Program, Dana-Farber Harvard Cancer Center, Boston, MA USA; 16 Department of Epidemiology, Harvard T.H. Chan School of Public Health, Boston, MA USA; 17 Broad Institute of MIT and Harvard, Cambridge, MA USA; 18 Department of Internal Medicine, University of Utah, Salt Lake City, Utah USA; 19 Department of Radiation Sciences, Oncology Unit, Umeå University, Umeå, Sweden; 20 Wallenberg Centre for Molecular Medicine, Umeå University, Umeå, Sweden; 21 Department of Medicine, Samuel Oschin Comprehensive Cancer Institute, Cedars-Sinai Medical Center, Los Angeles, CA USA; 22 Department of Preventive Medicine, Keck School of Medicine, University of Southern California, Los Angeles, CA USA; 23 Department of Laboratory Medicine and Pathology, Mayo Clinic, Rochester, MN USA; 24 Department of Pathology, School of Medicine, Umm Al-Qura University, Makkah, Saudi Arabia; 25 Department of Public Health Sciences, University of California Davis, Davis, CA USA; 26 Division of Gastroenterology, Massachusetts General Hospital and Harvard Medical School, Boston, MA USA; 27 Channing Division of Network Medicine, Brigham and Women’s Hospital and Harvard Medical School, Boston, MA USA; 28 Clinical and Translational Epidemiology Unit, Massachusetts General Hospital and Harvard Medical School, Boston, MA USA; 29 Broad Institute of Harvard and MIT, Cambridge, MA USA

**Keywords:** Genetic association study, Cancer genetics, Colorectal cancer

## Abstract

Identification of new genetic markers may improve the prediction of colorectal cancer prognosis. Our objective was to examine genome-wide associations of germline genetic variants with disease-specific survival in an analysis of 16,964 cases of colorectal cancer. We analyzed genotype and colorectal cancer-specific survival data from a consortium of 15 studies. Approximately 7.5 million SNPs were examined under the log-additive model using Cox proportional hazards models, adjusting for clinical factors and principal components. Additionally, we ran secondary analyses stratifying by tumor site and disease stage. We used a genome-wide p-value threshold of 5 × 10^–8^ to assess statistical significance. No variants were statistically significantly associated with disease-specific survival in the full case analysis or in the stage-stratified analyses. Three SNPs were statistically significantly associated with disease-specific survival for cases with tumors located in the distal colon (rs698022, HR = 1.48, CI 1.30–1.69, p = 8.47 × 10^–9^) and the proximal colon (rs189655236, HR = 2.14, 95% CI 1.65–2.77, p = 9.19 × 10^–9^ and rs144717887, HR = 2.01, 95% CI 1.57–2.58, p = 3.14 × 10^–8^), whereas no associations were detected for rectal tumors. Findings from this large genome-wide association study highlight the potential for anatomical-site-stratified genome-wide studies to identify germline genetic risk variants associated with colorectal cancer-specific survival. Larger sample sizes and further replication efforts are needed to more fully interpret these findings.

## Introduction

The global incidence of colorectal cancer (CRC) has been increasing while the mortality rate has been decreasing^1,2^. Advances in scientific knowledge, treatment modalities, and medical screening programs are considered among the major factors contributing to improved survival from this disease^1,3,4^. In the USA, Canada, Australia, and Europe, 5-year survival is around 65%^5–8^.

Currently, CRC prognostication relies primarily on clinicopathological features with a primary focus on tumor characteristics, such as stage. Several additional factors, in relation to both the tumor (e.g*. KRAS* and *BRAF* mutations) and the individual, have been associated with survival times and clinical outcomes^9–18^. Germline genetic variants are commonly investigated as candidate prognostic markers; they are abundant in the human genome, are polymorphic among patients, are thought to remain unchanged over time, and may biologically modify disease characteristics and risk of progression or clinical outcomes^19–21^. These characteristics of germline genetic variants, therefore, make them attractive for cancer research studies.

There is now a large body of research on genetic variants in relation to CRC incidence, yet no variants have been confidently associated with CRC survival or used in clinical practice. Many approaches have been attempted in survival outcomes studies, such as candidate SNP, gene, or pathway analyses^22,23^, including the examination of associations with patient outcomes for variants identified in susceptibility studies. Compared with other study designs, genome-wide association studies (GWAS) offer a comprehensive, agnostic approach. Some CRC survival GWAS have been performed^24–27^, which have identified a small number of genetic variants at the genome-wide significance level^25,26^. While these studies have advanced the knowledge of the genetic basis of CRC survival, they have also been limited by relatively small number of cases, restricting the ability to identify modest associations or low frequency risk variants, which may only be apparent when large case cohorts are examined.

In this study, we evaluated germline genetic loci associated with CRC-specific survival using data from 16,964 CRC participants included in an international consortium comprising 15 epidemiologic and clinical studies. As a secondary goal, we evaluated stage- and tumor site-specific associations between genetic loci and CRC survival.

## Results

Participant demographic and clinical characteristics are provided in Table 1. Median follow-up time after diagnosis was 13.8 years. Overall, 6,033 (36%) CRC cases died during follow up, of which 4,010 deaths (66%) were attributed to CRC. As expected, participants with stage 4 tumors at diagnosis were more likely to die from CRC (of those who died, 51% were stage 4 compared with 6% stage 1). Participants were ~ 50% female with a median age of 67 years (range 20–94 years).

**Table 1:** Demographics and tumor characteristics of 16,964 colorectal cancer patients.

	Overall (n = 16,964)		Died of CRC (n = 4,010)		Did not die of CRC (n = 12,954)	
	n	(%)	n	(%)	n	(%)
Age at diagnosis (median (range))	67	(20–94)	67	(24–94)	67	(20–94)
**Age category**						
< 50	984	(5.8)	263	(6.6)	721	(5.6)
50–60	2,751	(16.2)	705	(17.6)	2,046	(15.8)
60–70	6,520	(38.4)	1,435	(35.8)	5,085	(39.3)
> 70	6,709	(39.5)	1,607	(40.1)	5,102	(39.4)
All-cause deaths	6,033	(35.6)	4,010	(100.0)	2,023	(15.6)
CRC survival, years (median (IQR))	5.5	(3.40–9.77)	–	–	–	–
Overall survival, years (median (IQR))	6.1	(3.87–11.24)	–	–	–	–
Male Sex	8,528	(50.3)	2,045	(51.0)	6,483	(50.0)
**Stage**						
Stage 1 or local	3,338	(19.7)	157	(3.9)	3,181	(24.6)
Stage 2/3 or regional	6,420	(37.8)	1,209	(30.1)	5,211	(40.2)
Stage 4 or distant	1,847	(10.9)	1,448	(36.1)	399	(3.1)
Missing	5,359	(31.6)	1,196	(29.8)	4,163	(32.1)
**Tumor location**						
Proximal	6,214	(36.6)	1,433	(35.7)	4,781	(36.9)
Distal	4,881	(28.8)	978	(24.4)	3,903	(30.1)
Rectal	4,749	(28.0)	1,045	(26.1)	3,704	(28.6)
Missing	1,120	(6.6)	544	(13.8)	566	(4.4)
**Study**^**^**^						
CCFR	2,446	(14.4)	538	(13.4)	1,908	(14.7)
CPSII	819	(4.8)	186	(4.6)	633	(4.9)
DACHS	2,659	(15.7)	537	(13.4)	2,122	(16.4)
DALS	1,098	(6.5)	210	(5.2)	888	(6.9)
EDRN	191	(1.1)	14	(0.3)	177	(1.4)
EPIC	1,821	(10.7)	471	(11.7)	1,350	(10.4)
HPFS	348	(2.1)	85	(2.1)	263	(2.0)
MCCS	750	(4.4)	194	(4.8)	556	(4.3)
N9741	426	(2.5)	366	(9.1)	60	(0.5)
NHS	591	(3.5)	161	(4.0)	430	(3.3)
PHS	323	(1.9)	130	(3.2)	193	(1.5)
PLCO	972	(5.7)	174	(4.3)	798	(6.2)
UKB	2,919	(17.2)	581	(14.5)	2,338	(18.0)
VITAL	270	(1.6)	67	(1.7)	203	(1.6)
WHI	1,331	(7.8)	296	(7.4)	1,035	(8.0)

^**^**^*CRC* Colorectal Cancer, *CCFR* Colon Cancer Family Registry, *CPSII* Cancer Prevention Study II, *DACHS* Darmkrebs: Chancen der Verhütung durch Screening Study, *DALS* Diet, Activity and Lifestyle Study, *EDRN* Early Detection Research Network, *EPIC* European Prospective Investigation into Cancer, *HPFS* Health Professionals Follow-up Study, *IQR* interquartile range, *MCCS* Melbourne Collaborative Cohort Study, *NHS* Nurses’ Health Study, *PHS* Physicians' Health Study, *PLCO* Prostate, Lung, Colorectal and Ovarian Cancer Screening Trial, *UKB* UK Biobank, *VITAL* Vitamins and Lifestyle, *WHI* Women’s Health Initiative.

**Figure 1:**
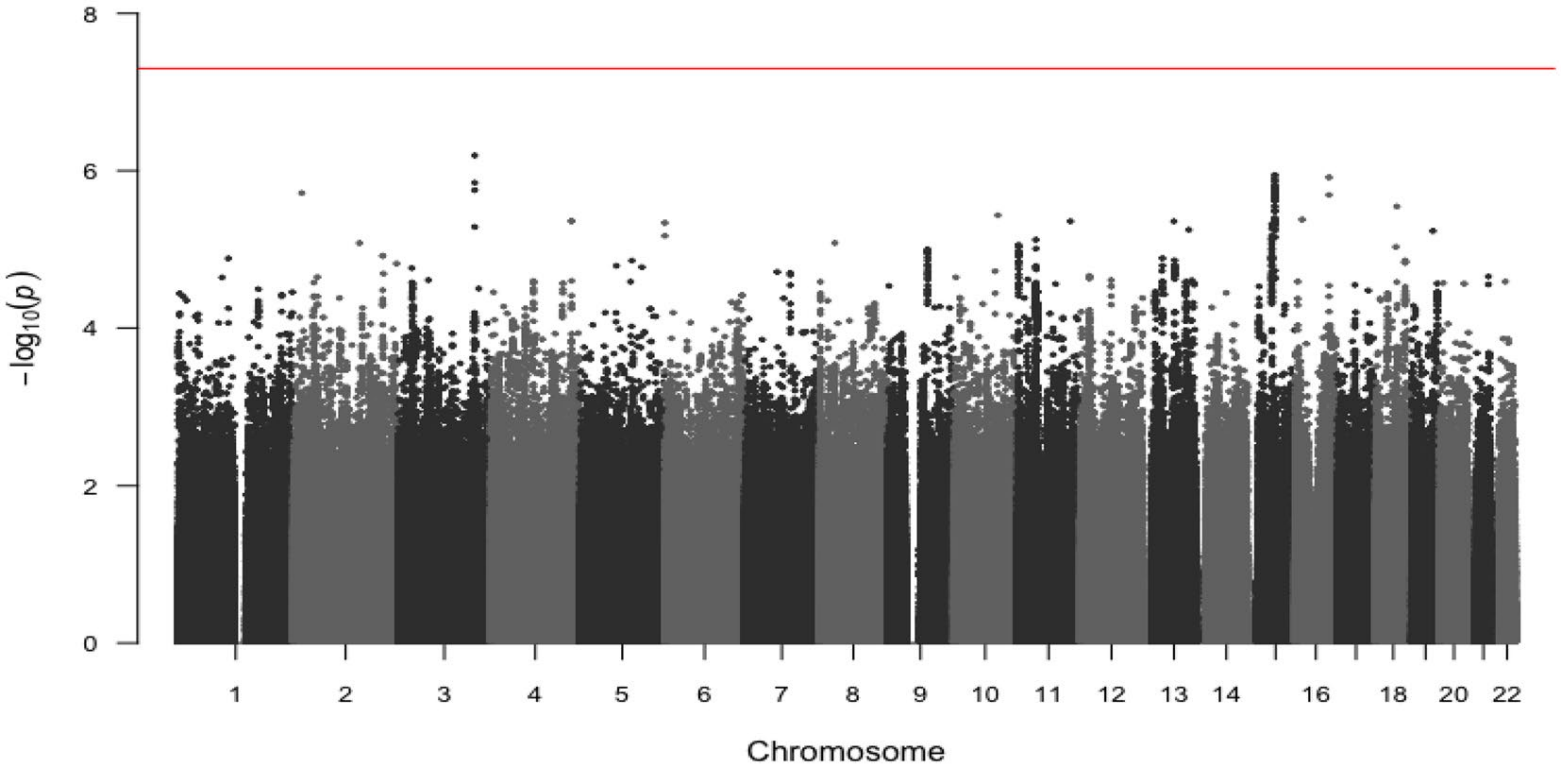
Manhattan plot of -log_10_ p-values by genomic position for the genome-wide analysis of colorectal cancer survival in 16,964 cases. The red line indicates genome-wide significance threshold (p = 5 × 10^–8^).

**Figure 2:**
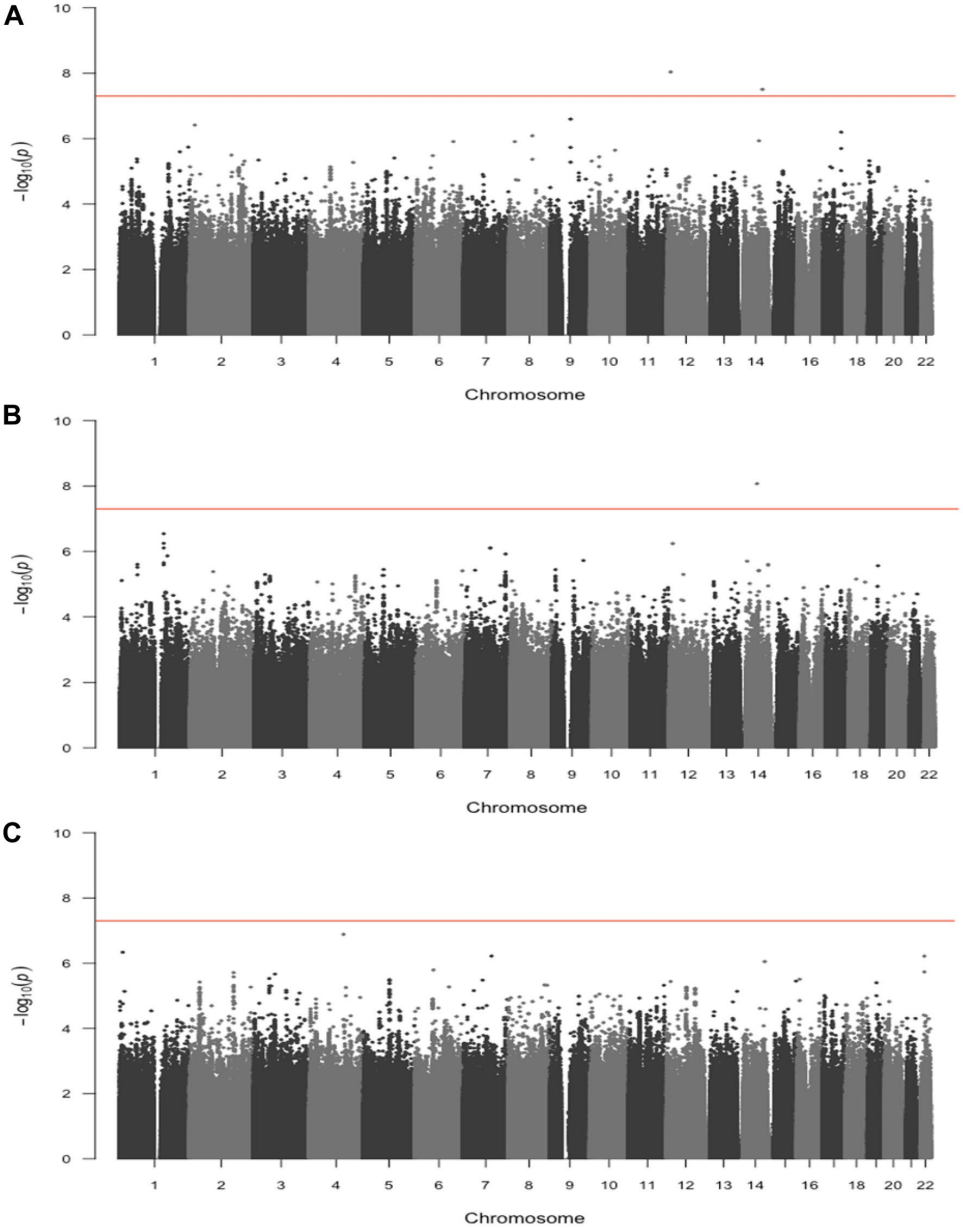
Manhattan plots of -log_10_ p-values by genomic position for the genome-wide analysis of colorectal cancer survival stratified by tumor site. (**A**) proximal colon tumor-specific in 6,214 cases, (**B**) distal colon tumor-specific in 4,881 cases, (**C**) rectal tumor-specific in 4,749 cases. The red line indicates genome-wide significance threshold (p = 5 × 10^–8^).

No substantial systemic inflation was identified from quantile-quantile (QQ) plots (Supplementary Fig. 1). No variants reached genome-wide significance for the primary GWAS (Fig. 1) or for the stage-stratified analysis (Supplementary Fig. 2, Supplementary Table 3). Two variants were statistically significant at a genome-wide *P*-value threshold among proximal colon tumors and one among distal colon tumors (Table 2, Fig. 2). No variants reached genome-wide significance among rectal tumors. The significant variants identified among proximal colon tumors were located on chromosomes 12 (rs189655236, hazard ratio [HR] = 2.14, 95% confidence interval [CI]: 1.65–2.77, p = 9.19 × 10^–9^) and 14 (rs144717887, HR = 2.01, 95% CI: 1.57–2.58, p = 3.14 × 10^–8^). Both variants were low frequency (MAF 1.4% and 1.5%, respectively) and neither were in linkage disequilibrium (LD; defined as R^2^ > 0.6) with nearby variants. The rs189655236 variant was located within the intronic region of *BORCS5* and rs144717887 was located in an intergenic region. The variant significantly associated with CRC survival among distal colon tumors was located in an intergenic region on chromosome 14 (rs698022, HR = 1.48, CI 1.30–1.69, p = 8.47 × 10^–9^), was common with a minor allele frequency (MAF) of 11%, and was not in LD (R^2^ < 0.6) with nearby variants. The two chromosome 14 variants identified in proximal and distal colon analyses were not in linkage disequilibrium with each other. None of the statistically significant variants were predicted to have regulatory effects (ranking scores = 4 or 5) as reported in the RegulomeDB database.

**Table 2:** Variants associated with colorectal cancer survival at* P* < 5 × 10^–8^, stratified by tumor site.

Chromosome	Variant rsID	Alleles (risk/alternative)	RAF	RegulomeDB rank	HR^a^	95% CI	P-value	Imputation quality (info score)
**Proximal colon**								
12	rs189655236	C/T	0.014	5	2.14	(1.65, 2.77)	9.19 × 10^–09^	0.85
14	rs144717887	G/A	0.015	5	2.01	(1.57, 2.58)	3.14 × 10^–08^	0.93
**Distal colon**								
14	rs698022	C/T	0.111	4	1.48	(1.30, 1.69)	8.47 × 10^–09^	0.93

Proximal colon tumor-specific analyses included 6,214 cases and distal colon tumor-specific analyses included 4,881 cases.

*RAF* Risk Allele Frequency, *HR* Hazard Ratio, *CI* Confidence Interval. ^a^Adjusted for age at diagnosis, sex, genotyping batch/study, and the first five principal components of genetic ancestry.

## Discussion

In this analysis of common genetic variants in a sizeable study population, we did not identify any SNPs associated with CRC prognosis at the genome-wide significance level. We also found no SNPs associated with survival for specific tumor stages. However, our results suggest that there may be variants that predict CRC-survival for distal and proximal colon cancer cases.

The distal and proximal regions of the colon differ biologically and in terms of tumor incidence rates^28,29^. In addition, research shows that tumors located in these anatomical subsites display differences in molecular alterations involved in tumorigenesis, and are characterized by different disease progression and prognosis^30–32^. The identification of different sets of variants with survival for cases with distal and proximal colon tumors in this study is, therefore, not surprising. Using eQTLGen, rs189655236 was predicted to be in *cis-*eQTL with DUSP16, which has been associated with chemotherapy resistance in colorectal cancer^33^. However, none of the identified SNPs were predicted to have putative regulatory functions using RegulomeDB, and according to the dbSNP database, they are within intronic or intergenic sequences^34^. Also, the two significant variants for proximal colon tumors are very low frequency. These considerations lead us to interpret our findings with caution pending further investigation.

This study has several strengths. By leveraging data from 15 population-based and clinical studies, we were able to confidently examine with good statistical power genetic associations with CRC survival. Covariates were well characterized with detailed information on epidemiologic and clinical factors which allowed us to conduct subgroup analysis by stage at diagnosis and tumor anatomical location. In addition, we had a relatively long follow-up period and cause of deaths were uniformly ascertained. We also used an agnostic discovery-based approach to identify variants associated with CRC survival.

Our study also has some limitations. Although we had a large enough sample size to identify significant SNPs in CRC cases with European ancestry, we were unable to evaluate other ancestry groups. Additionally, we were unable to evaluate other tumor markers that might be associated with survival in our population. Another limitation inherent to the GWAS approach is the high likelihood of false-negative findings due to the stringent *P*-value threshold for genome-wide significance. This threshold is set to account for multiple testing and is designed to reduce the number of false-positive findings; however, a consequence of this stringency is that some important SNP-survival associations may have been missed. Finally, no replication analysis or functional follow-up was conducted.

In summary, in this largest yet GWAS for CRC specific survival, our analyses indicate that genetic variants in the form of SNPs are unlikely to explain variable risk of death from colorectal cancer in people of European ancestry. However, a few SNPs were identified that may be prognostic markers for distal or proximal colon cancers and these should be further examined in other populations, including cases from other ancestry groups.

## Methods

### Study population

Analyses utilized data from the International Survival Analysis in Colorectal Cancer Consortium (ISACC), a compilation of participants with incident, invasive CRC obtained from clinical trials, case–control, and cohort studies from around the world. Study participants included people of European genetic ancestry diagnosed with invasive CRC and with available genotyping and CRC-specific survival data (as described in the Supplementary Methods). The following 15 ISACC studies were included: the Cancer Prevention Study-II (CPS-II)^35^, the German Darmkrebs: Chancen der Verhutung durch Screening Study (DACHS)^36^, the Diet Activity and Lifestyle Study (DALS)^37^, the Early Detection Research Network (EDRN)^38^, the European Prospective Investigation into Cancer (EPIC)^39^, the Health Professionals Follow-up Study (HPFS)^40^, the Melbourne Collaborative Cohort Study (MCCS)^41^, the Nurses’ Health Study (NHS)^42,43^, the N9741 clinical trial^44^, the Physician’s Health Study (PHS)^45,46^, the Prostate, Lung, Colorectal, and Ovarian Study (PLCO)^47,48^, the UK Biobank (UKB)^49^, the VITamins And Lifestyle Study (VITAL)^50^, the Women’s Health Initiative (WHI)^51,52^, and four Colon Cancer Family Registry (CCFR) sites^53,54^: Seattle, Ontario, Australia, and the Mayo Clinic. Study-specific details are described in the Supplementary Tables 1 and 2.

### Ethical considerations

Study protocols were approved by the Institutional Review Board or Independent Ethics Committee overseeing the respective clinical sites. Participants provided informed consent for genetic testing and research participation. The study protocol has been approved by Fred Hutchinson Cancer Research Center Institutional Review Board. All methods were performed in accordance with the relevant guidelines and regulations.

### Ascertainment of CRC-specific survival

Protocols for survival outcomes assessment in this study population have been described previously^35–38,43–45,49,50,53,55–61^. Briefly, studies ascertained vital status via linkage to the National Death Index, state cancer registries, state death records, or population registers with cause of death verified by death certificates (CPSII, DACHS, DALS, EPIC, MCCS, UKB, VITAL), or via active follow-up (CCFR, HPFS, NHS, PHS, PLCO, WHI, N9741) with dates and cause of death confirmed via regional mortality databases, review of death certificates and/or medical records by trained adjudicators. In all studies, cases alive at the most recent study follow-up or data linkage were censored on that date. In VITAL, individuals who moved outside of Washington State were censored at their date of move. CRC-specific survival was calculated as days from diagnosis to CRC-related death or end of follow-up. Individuals who died from causes other than CRC were censored at the time of death.

### Tumor stage and location classification

Tumor stage was obtained from pathology and registry reports at the time of diagnosis. The Surveillance, Epidemiology, and End Results (SEER) summary stage categorizations of localized, regional, and distant were used, also incorporating extent of disease information when available. Additionally, the American Joint Committee on Cancer (AJCC) TNM classification of malignant tumors (TNM) categorizations were utilized to assign values I through IV.

Tumor location was obtained from registry and pathology reports. Location was grouped based on ICD-9 codes as follows: (1) “Proximal” (153.0/Hepatic flexure, 153.1/Transverse colon, 153.4/Cecum, 153.6/Ascending colon), (2) “Distal” (153.2/Descending colon, 152.3/Sigmoid colon, 153.7/Splenic flexure), 3) “Rectal” (154.0/Rectosigmoid junction, 154.1/Rectum).

### Genotype data

Genotyping methods have been reported previously^62–66^. Briefly, genomic DNA was extracted from blood or buccal samples using conventional methods, and samples were genotyped using the platforms listed in Supplementary Table 1. Each genotyping platform dataset underwent standard quality control analyses, including exclusion of samples and SNPs with low call rates (< 97% and < 98%, respectively), exclusion of variants departing from Hardy–Weinberg Equilibrium (p < 1 × 10^–4^), exclusion of individual with discrepant reported and genotyped sex based on X chromosome heterozygosity, and exclusion of duplicates and individuals that were second-degree or more closely related based on identity by descent (IBD) calculations. Additionally, we inferred genetic ancestry using principal components analysis and excluded individuals of non-European ancestry from analyses due to small sample sizes. Participants with a value within one standard deviation of the median for the first and second eigenvectors were categorized as European genetic ancestry and included in the analysis (Supplementary Methods). A total of 16,964 individuals passed quality control filtering. Only variants passing quality control analyses and with missing call rates ≤ 2% were used for imputation.

Phasing and imputation were performed on each pooled set of studies with the same or similar genotyping platforms. Autosomal variants were phased using SHAPEIT2 and imputed to the Haplotype Reference Consortium panel release 1.1 (~ 39 million variants) using the University of Michigan Imputation Server^67–69^. Genotype probabilities were converted to allelic dosages. Evaluation was restricted to variants with MAF ≥ 1% and imputation accuracy R^2^ > 0.3. A total of 7,829,749 genetic variants were included in the analyses. All imputed and cleaned individual-level genotype data were pooled for survival analyses.

We used PLINK (v1.9) for principal components analysis on pruned sets of autosomal variants obtained by removing regions with extensive long-range linkage disequilibrium. The first five principal components were used as coviariates to account for population substructure in analysis.

### Statistical analysis

We used Cox proportional hazards regression to estimate HRs and 95% CIs for associations of each genetic variant with CRC-specific survival. A log-additive model was used, relating variant genotype dosage to CRC-specific survival. All models were adjusted for age at diagnosis, sex, a categorical variable encompassing genotyping platform and study, and five principal components to account for population substructure. The proportional hazards assumptions for age and sex were evaluated by testing for a non-zero slope of the scaled Schoenfeld residuals on ranked failure time^70^. The tests for both age and sex were statistically significant (p < 0.05), suggesting the proportionality assumption may not hold. For age, the non-proportionality was resolved by including both a continuous and categorical variable (dichotomized at median age; ≤ 57 years versus > 57 years). We stratified our analysis by sex.

As a secondary goal, we evaluated the association of genetic loci and CRC survival stratified by tumor stage at diagnosis and anatomical location. Tumor stage strata included regional (stages 2 and 3) and distant metastatic (stage 4) disease; local (stage 1) disease was not evaluated due to a low percentage of deaths among this group. Tumor anatomical location was grouped as “proximal colon” (ICD-9-CM 153.0/Hepatic flexure, 153.1/Transverse colon, 153.4/Cecum, 153.6/Ascending colon), “distal colon” (153.2/Descending colon, 152.3/Sigmoid colon, 153.7/Splenic flexure), or “rectum” (154.0/Rectosigmoid junction, 154.1/Rectum).

We evaluated QQ plots of log-transformed p-values and calculated genomic control coefficients to assess for possible systemic inflation. We produced Manhattan plots and specified a genome-wide statistical significance level of p ≤ 5 × 10^–8^. We performed statistical analyses using R version 3.5.2.

### In silico analyses

The NCI ‘LDassoc’ web tool (https://ldlink.nci.nih.gov/) was used to evaluate LD (defined as R^2^ > 0.6) in 1000 Genomes Phase 3 ‘EUR’ population) for SNPs of interest^71^. The putative functional effects of variants were inferred based on information in the RegulomeDB database^72^. This database ranks variants ranging from 1–7 such that lower ranks represent variants with greater predicted regulatory impact (https://regulomedb.org/regulome-help/). For example, eQTLs (expression quantitative trait loci) have a rank of 1; variants locate in a transcription factor binding motif and DNAse peak have a rank of 4; and variants that locate in a transcription factor binding motif or a DNAse peak have a rank of 5. We additionally assessed *cis*-eQTLs using the eQTLGen Consortium database (https://www.eqtlgen.org/). ^73^.

## Supplementary Information

Supplementary Information.
