# Supplementary material for: Genome-wide association study identifies tumor anatomical site-specific risk variants for colorectal cancer survival
Source: Sci Rep. 2022 Jan 7;12:127. doi: 10.1038/s41598-021-03945-x (PMC8741984; doi:10.1038/s41598-021-03945-x)
Supplement: Supplementary file 1 — Supplementary Information. [file 41598_2021_3945_MOESM1_ESM.docx]

Supplementary Material

Table of Contents

[Supplementary Methods 2](#_Toc84875943)

[Classification of European Genetic Ancestry 2](#_Toc84875944)

[Supplementary Tables 3](#_Toc84875945)

[Supplementary Table 1. Study-specific methodology. 3](#_Toc84875946)

[Supplementary Table 2. Demographics and tumor characteristics of 16,964 colorectal cancer patients, stratified by study. 4](#_Toc84875947)

[Part 1 of 2 4](#_Toc84875948)

[Part 2 of 2 5](#_Toc84875949)

[Supplementary Table 3. Hazard ratios, 95% confidence intervals, and p-values for rs189655236, rs698022, and rs144717887 in overall GWAS and all subgroup analyses. Bolded results met our threshold for genome-wide significance. 6](#_Toc84875950)

[Supplementary Figures 7](#_Toc84875951)

[Supplementary Figure 1. Quantile-quantile (QQ) plots of -log_10_ transformed p-values for variant-specific associations with colorectal cancer survival. Grey shading indicates the 95% CIs based on the beta distribution of observed p-values. (A) overall GWAS, (B) stage 2/3-specific, (C) stage 4-specific, (D) proximal colon tumor-specific, (E) distal colon tumor-specific, (F) rectal tumor-specific. 7](#_Toc84875952)

[Supplementary Figure 2. Manhattan plot of -log_10_ p-values for the genome-wide analysis of colorectal cancer survival stratified by tumor stage. (A) stage 2/3-specific, (B) stage 4-specific 8](#_Toc84875953)

[Supplementary Figure 3. Principal components analysis (PCA) used to define European genetic ancestry. (A) PCA plot colored by self-identified race. Values within one standard deviation of the median for the first and second eigenvectors was used to define individuals with European genetic ancestry. (B) individuals defined as European genetic ancestry are highlighted in blue. All others (n=1,220) were excluded from further analysis. 9](#_Toc84875954)

# Supplementary Methods

## Classification of European Genetic Ancestry

To reduce confounding by genetic ancestry and due to relatively small sample sizes of non-White cases in the study populations, we restricted the analysis to participants with European genetic ancestry, as determined by principal components analysis (PCA). PCA was performed on the full ISACC study population using PLINK (v1.9). The first two eigenvectors classified individuals based on self-identified race (Supplementary Figure 3A) and were used to categorize participants by European genetic ancestry. Participants with a value within one standard deviation of the median for the first and second eigenvectors were categorized as European genetic ancestry (Supplementary Figure 3B) and retained for subsequent analyses.

# Supplementary Tables

## Supplementary Table 1. Study-specific methodology.

*Registry linkage involves National Death Index, state cancer registries, state death records, or population registers with cause of death verified by death certificates; Active follow-up involves death certificate and/or medical record review.

## Supplementary Table 2. Demographics and tumor characteristics of 16,964 colorectal cancer patients, stratified by study.

### Part 1 of 2

### Part 2 of 2

## Supplementary Table 3. Hazard ratios, 95% confidence intervals, and p-values for rs189655236, rs698022, and rs144717887 in overall GWAS and all subgroup analyses. Bolded results met our threshold for genome-wide significance.

# Supplementary Figures


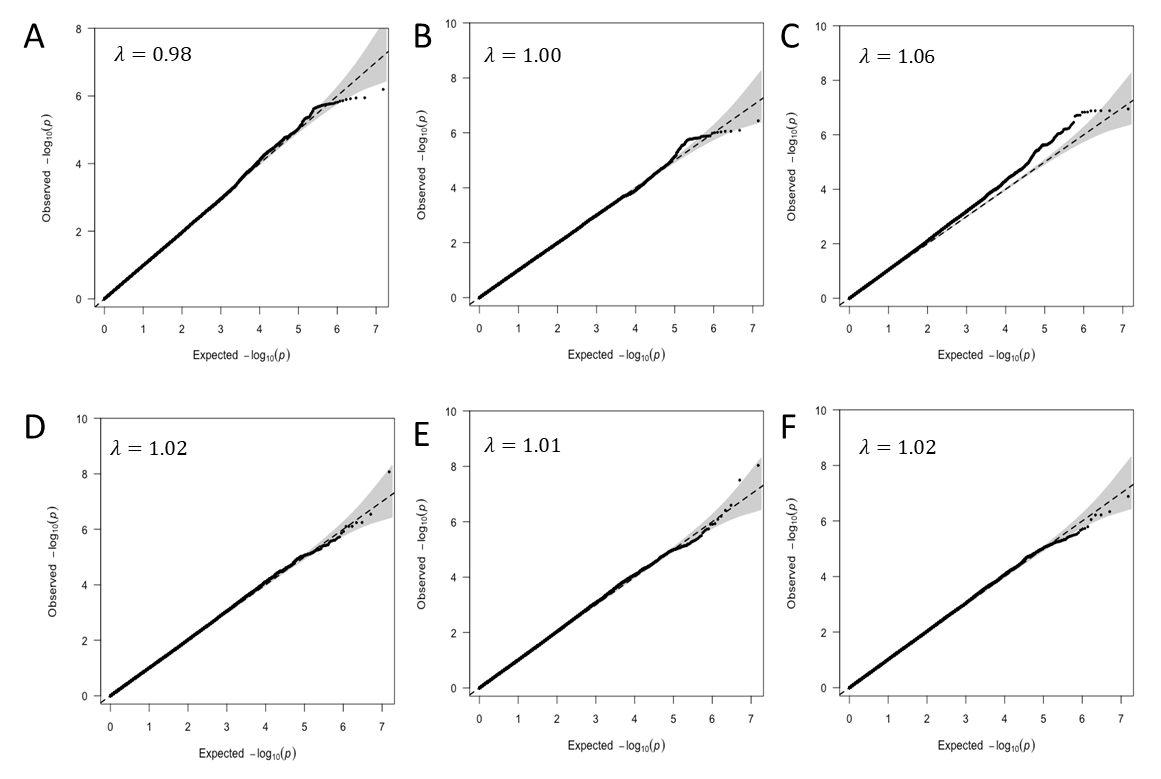


## Supplementary Figure 1. Quantile-quantile (QQ) plots of -log_10_ transformed p-values for variant-specific associations with colorectal cancer survival. Grey shading indicates the 95% CIs based on the beta distribution of observed p-values. (A) overall GWAS, (B) stage 2/3-specific, (C) stage 4-specific, (D) proximal colon tumor-specific, (E) distal colon tumor-specific, (F) rectal tumor-specific.


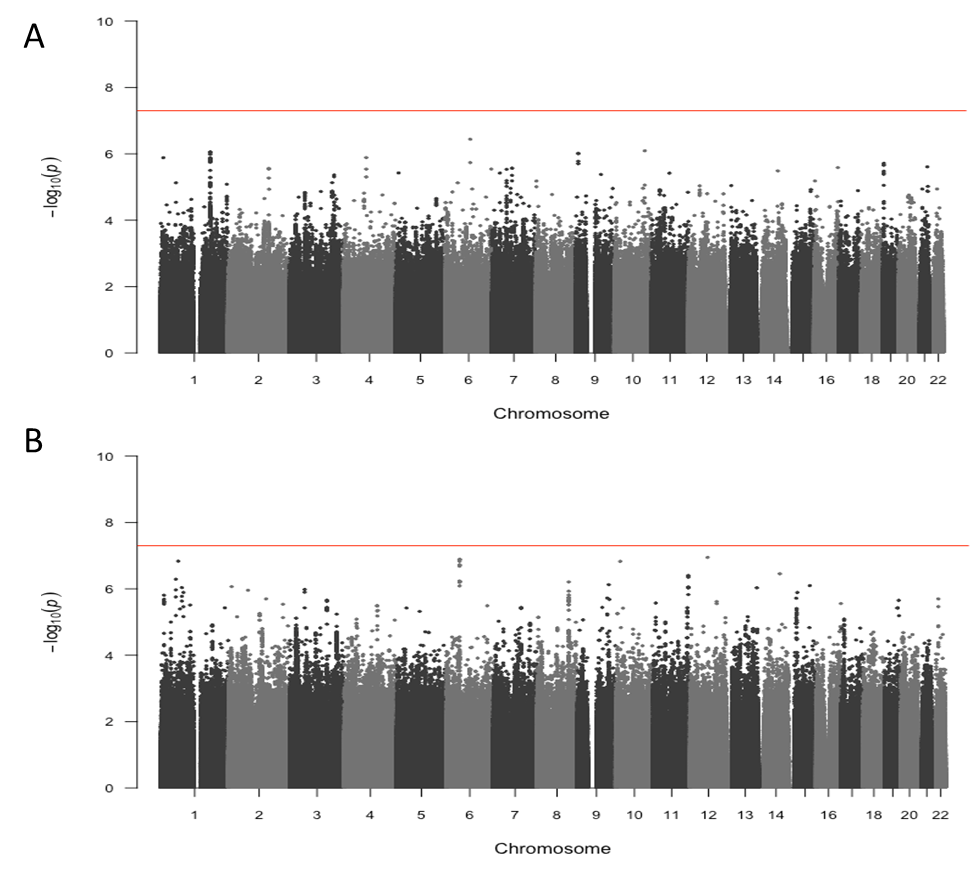


## Supplementary Figure 2. Manhattan plot of -log_10_ p-values for the genome-wide analysis of colorectal cancer survival stratified by tumor stage. (A) stage 2/3-specific, (B) stage 4-specific


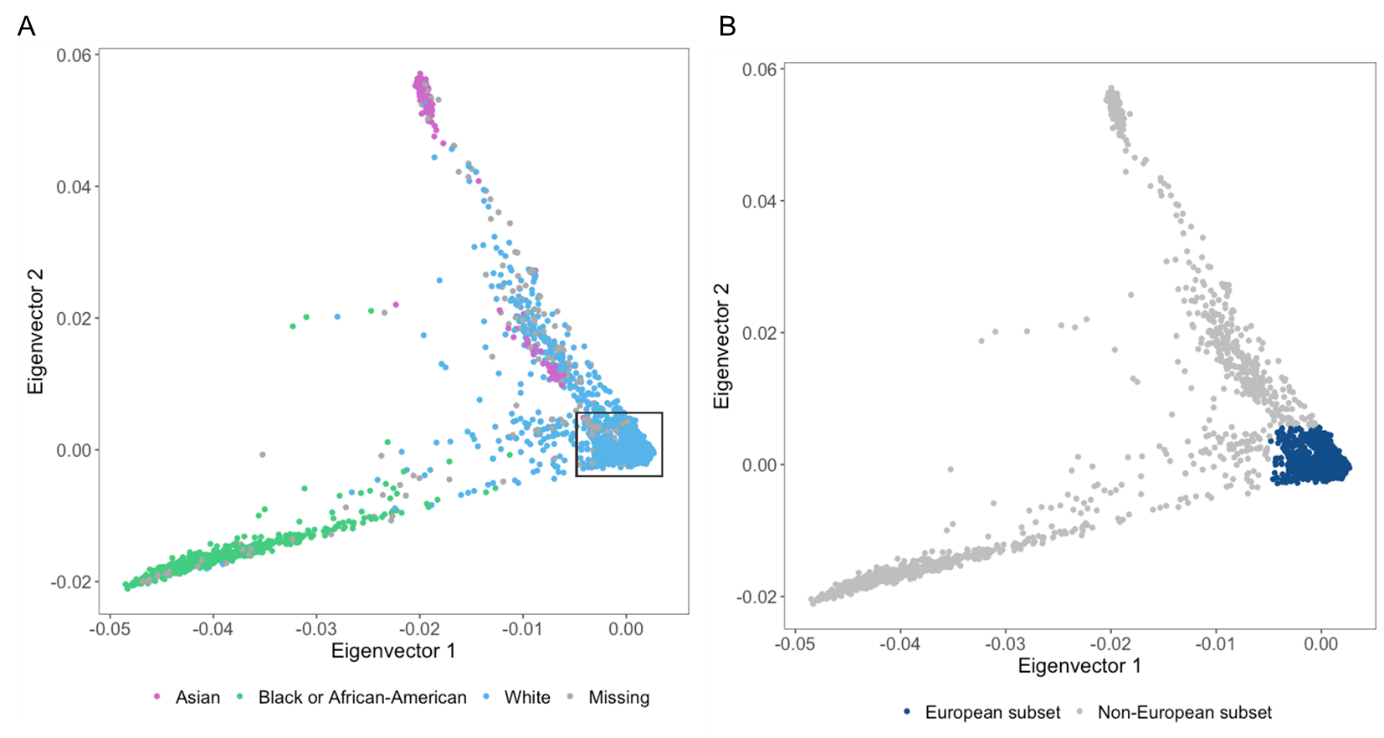


## Supplementary Figure 3. Principal components analysis (PCA) used to define European genetic ancestry. (A) PCA plot colored by self-identified race. Values within one standard deviation of the median for the first and second eigenvectors was used to define individuals with European genetic ancestry. (B) individuals defined as European genetic ancestry are highlighted in blue. All others (n=1,220) were excluded from further analysis.
